# Generation, Purification and Transplantation of Photoreceptors Derived from Human Induced Pluripotent Stem Cells

**DOI:** 10.1371/journal.pone.0008763

**Published:** 2010-01-20

**Authors:** Deepak A. Lamba, Andrew McUsic, Roli K. Hirata, Pei-Rong Wang, David Russell, Thomas A. Reh

**Affiliations:** 1 Department of Biological Structure, University of Washington, Seattle, Washington, United States of America; 2 Department of Medicine, University of Washington, Seattle, Washington, United States of America; 3 Department of Biochemistry, University of Washington, Seattle, Washington, United States of America; 4 Institute for Stem Cells and Regenerative Medicine, University of Washington, Seattle, Washington, United States of America; Universidade Federal do Rio de Janeiro (UFRJ), Brazil

## Abstract

**Background:**

Inherited and acquired retinal degenerations are frequent causes of visual impairment and photoreceptor cell replacement therapy may restore visual function to these individuals. To provide a source of new retinal neurons for cell based therapies, we developed methods to derive retinal progenitors from human ES cells.

**Methodology/Physical Findings:**

In this report we have used a similar method to direct induced pluripotent stem cells (iPS) from human fibroblasts to a retinal progenitor fate, competent to generate photoreceptors. We also found we could purify the photoreceptors derived from the iPS cells using fluorescence activated cell sorting (FACS) after labeling photoreceptors with a lentivirus driving GFP from the IRBP cis-regulatory sequences. Moreover, we found that when we transplanted the FACS purified iPSC derived photoreceptors, they were able to integrate into a normal mouse retina and express photoreceptor markers.

**Conclusions:**

This report provides evidence that enriched populations of human photoreceptors can be derived from iPS cells.

## Introduction

Retinal degenerations that involve rod and cone photoreceptors are a major cause of blindness, and affect millions of people in the US. These devastating conditions can be inherited or acquired, and while efforts are underway to develop treatments that slow or prevent these conditions using gene therapy or medical treatments, once the photoreceptors have degenerated, cell replacement or prosthetic devices are the only options. Cell replacement of photoreceptors has been shown to be feasible, even in mature mice, where photoreceptors transplanted to the sub-retinal space can integrate into the retina and function [1], [2]. We, and others, have developed protocols for generating retinal progenitors and photoreceptors from human embryonic stem cells as a potential source of replacement photoreceptors for cell-based therapy of retinal degenerations [3], [4], [5]. Our protocol involves the directed differentiation of undifferentiated human embryonic stem (hES) cells into retinal progenitor cells, followed by expansion of these cells and their differentiation into photoreceptors. These cells can be transplanted to the sub-retinal space of visually deficient mice and can restore some light response [2].

One of the problems with cell-based therapies is that recipients may require immuno-suppressant drugs to prevent rejection of the transplanted cells. One way around this complication is to use cells derived from closely related or HLA-matched individuals or even the patients themselves, using induced pluripotent stem cells (iPS). iPS cells were initially generated by Shinya Yamanaka and colleagues by expressing combinations of candidate genes into mouse embryonic fibroblasts [6]. The four genes that were required, *OCT3/4, SOX2, KLF4* and *c-MYC* have also been shown to induce pluripotency in human fibroblasts, and various combinations of these and small molecules can efficiently induce the pluripotent state in a variety of different cell types [6], [7], [8], [9], [10], [11]. The iPS cells behave similarly to ES cells in most assays, including contributing to mouse germline transmission [9]. Several groups have shown that iPS cells can be directed to a variety of lineages and may be useful for studying specific diseases where animal models do not exist or are inadequate (eg. [12]).

The similarity of iPS cells to hES cells led us to ask whether these cells would respond to our retinal determination protocol like the ES cells. In this report we show that iPS cells, generated with the combination of *OCT4, SOX2, NANOG* and *LIN28*, can be directed to a retinal progenitor fate using the same protocol as we developed for hES cells. Moreover, we show for the first time that iPSC-derived photoreceptors can be identified in the cultures by infection with a viral construct in which GFP is driven from a photoreceptor-specific (IRBP) promoter. The GFP+ photoreceptors can be highly purified using FACS, providing a potentially unlimited source for cell-based therapies of retinal degenerations. Lastly, we show that the iPSC-derived photoreceptors that have been purified by FACS integrate into the outer nuclear layer after transplantation to the sub-retinal space of adult mice, similar to normal mouse photoreceptors and ES cell derived rod photoreceptors. The ability to derive retinal photoreceptors from iPS cells will also be useful in the development of in vitro models of specific human retinal degenerations.

## Materials and Methods

### Cell Culture and Retinal Induction

H-1 (WA01) line was obtained from Wicell, the Hues6, Hues14 and Hues16 were obtained from Doug Melton (Harvard University, MA) and Mel1 and Mel2 lines were purchased from Millipore. An iPS cell line was made by infecting a human fibroblast culture (Coriell GM05387) with lentiviral vectors expressing OCT4, NANOG, LIN28 and SOX2 as described [11]. All cell lines were initially maintained with CF-1 feeders and subsequently adapted to feeder-free conditions. Retinal induction was performed by modifying the protocol previously described [5]. Instead of making embryoid bodies, 25–30 of the 150–200 ES cell clumps were plated on Matrigel coated 35mm dishes and cultured for 3 days in the presence of mouse noggin (1 ng/ml, R&D Systems), human recombinant Dkk-1 (1 ng/ml, R&D Systems) and human recombinant insulin-like growth factor-1 (IGF-1) (1 ng/ml, R&D Systems). From the fourth day, the concentration of the factors was increased to 10 ng/ml. The media was changed every 2–3 days for up to three weeks. The cells were cultured for several months in media containing N2 and B27 supplement without any additional factors for further differentiation.

### Immunocytochemistry and Immunohistochemistry

Cells and eyes were fixed with 4% paraformaldehyde and analyzed with the following antibodies using protocol previously described: rabbit anti-recoverin (gift from Dr. Jim Hurley, University of Washington, 1∶1000), mouse anti-Hu C/D (Molecular Probes, 1∶200), mouse anti-rhodopsin (Rho-4D2) (gift from Dr. Molday, University of British Columbia, 1∶750), mouse anti-Pax6 (1∶250) and mouse anti-SSEA4 (1∶10) (DHSB), mouse anti-human α-SMA, mouse anti-human AFP and mouse anti-MAP2 (Millipore), chicken anti-GFP (1∶500) and rabbit anti-Sox9 (1∶400) (Abcam), rabbit anti-S-opsin (gift from Dr. J Nathans, Johns Hopkins University, 1∶1000), rabbit anti-Nrl (gift from Dr. A. Swaroop, 1∶500), goat anti-Otx2-biotin (R&D Systems, 1∶250), rabbit anti-AIPL1 (gift from Dr. V. Ramamurthy, 1∶5000), rabbit anti-Crx (gift from Dr. C. Craft, 1∶250), rabbit anti-ZO-1 (Invitrogen, 1∶250), goat anti-Sox2 (1∶500), goat anti-Oct3/4 (1∶250), goat anti-Brn3 (1∶100) and rat anti-Blimp1 (1∶100) (Santa Cruz). Secondary antibody staining was done using the corresponding Cy5 (Jackson Immunoresearch), Cy5-conjugated streptavidin, Alexa-488, Alexa-568, and Alexa 633 fluorescent-conjugated antibodies (Invitrogen, 1∶500). The primary antibodies were used at the appropriate dilution in 0.5% Triton X-100 and 5% dry milk in PBS overnight at 4°C. The slides were then washed 3 times in PBS followed by 1 hour in secondary antibody at room temperature. Images were taken using a Nikon A1 confocal microscope. Image analysis was performed using Volocity software (Improvision) and Adobe Photoshop CS4. All counts shown as mean +/− SEM.

### Virus Production and Infection

EF-1a-GFP lentivirus was made using constructs provided by Dr. Charles Murry (University of Washington). The IRBP-GFP lentivirus was prepared using the human IRBP promoter from Dr. Paul Overbeek (Baylor College of Medicine, TX). pRRL-cPPT-CMV lentivirus plasmid was cut to remove the CMV promoter and replaced with the multiple cloning site and the eGFP portion from pEGFP1 plasmid (Clonetech). The human IRBP promoter was next subcloned into the multiple cloning site to drive eGFP expression. Both lentiviruses are 3rd generation replication-incompetent lentivirus and were made using the four-plasmid system as previously described [2]. The ES cultures were infected with either the EF-1a lentivirus or the IRBP-GFP lentivirus from four to eight weeks after the induction of retinal determination, and were maintained for an additional one to two weeks to allow for expression of the GFP.

### Fluorescent Activated Cell Sorting (FACS)

To isolate the cells that express IRBP-GFP from the ES cultures, we dissociated them into a single-cell suspension using trypsin. Prior to sorting, aggregates were removed by passing through a 40 µm cell strainer. FACS was carried out on BD Aria II sorter, gated for a high level of GFP expression.

### Explant Culture

Retinas from newborn mouse, embryonic day 4 chicken, and 82 day, and 90 96 day fetal human retinas (obtained from the Laboratory of Developmental Biology - NIH HD 000836 at the University of Washington without identifiers) were dissected from extra-ocular tissues and cultured in the presence of IRBP-GFP lentivirus as free-floating explants in ultra-low attachment plates overnight. The media was then changed and the explants were maintained for an additional four days, prior to fixation with 4% PFA and cryosectioning. One of the fetal human retinas was fixed for cryosectioning while the other was trypsinized and used for FACS and microarray analysis.

### Real Time Quantitative PCR Analysis

Total RNA was extracted from the cultures using TriZol (Invitrogen) followed by chloroform extraction as per manufacturer's instructions. This was followed by DNAse-1 (Qiagen) treatment followed by RNA cleanup using Qiagen RNA mini cleanup kit. cDNA was reverse transcribed using Superscript II RT kit (Invitrogen) as per manufacturer's instructions. Q-PCR was performed for various genes as previously described [5] and results normalized to β−actin levels.

### Microarray Analysis

For microarray analysis, a 96 day human fetal retina and FACS purified human fetal photoreceptors were lyzed in Trizol and RNA extracted as described above. The RNA was then checked for RNA integrity and run as per manufacturer's guidelines on the Human Gene 1.0 ST chip (Affymetrix). The data was then normalized in the GCOS software and was then analyzed using Multi-Experiment Viewer (v. 4.4) software.

### Cell Transplantation

All experiments were done in accordance with approved protocols and the animals were housed and bred in the Department of Comparative Medicine at the University of Washington. To test whether the iPSC-derived photoreceptors could integrate into normal retina, adult wild-type mice were anesthetized and approximately 50,000 FACS purified iPSC derived photoreceptors were injected into the sub-retinal space as previously described [2]. Animals that received injections of iPSC derived photoreceptors also received daily injections of the immunosuppressant Cyclosporine A (10 mg/kg/day). After survival periods of two to three weeks, the animals were sacrificed, the retinas removed, cryosectioned and processed for immunofluorescent analysis. To determine whether the undifferentiated iPSCs could generate teratomas, we carried out the assays as described in a previous publication [13].

## Results

**Figure 1:**
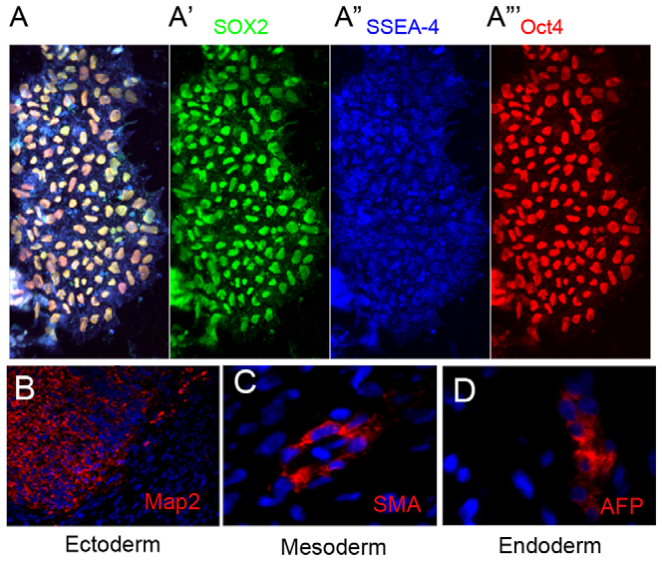
Co-expression of undifferentiated cell markers Sox2 (green, A′), SSEA-4 (blue, A″) and Oct4 (red, A″′) by iPS cells with merged view in (A). (B-D) Tri-lineage potential of teratomas formed by iPSC-MHF2 c1 in immunodeficient mice. Histological sections from a teratoma stained with antibodies against human MAP-2 (microtubule associated protein-2, B), human SMA (alpha smooth muscle actin, C), or human AFP (alpha-fetoprotein) and co-stained with DAPI (blue).

The iPS cells were generated from normal human fibroblast cultures as described previously (Yu et al., 2007) with lentiviral vectors expressing OCT4, NANOG, LIN28 and SOX2. Upon quadruple infection, several human ESC-like colonies appeared after three weeks and were isolated and expanded further. One particular clone (iPSC-MHF2 c1) was chosen for our experiments. When cultured, these cells expressed the pluripotency markers SOX2, OCT4 and SSEA-4 (figure 1A-A″′). The cells could be maintained with CF-1 feeder layers as well as in feeder-free MEF-conditioned media. In vivo, the iPS cells were capable of differentiation into cells of all three germ layers upon teratoma formation in immunodeficient mice (Figure 1B, C, D), confirming that the iPSCs were pluripotent. Additionally, we looked for silencing of exogenous pluripotency genes and found that even though Nanog and Lin28 were silenced, there was still some expression of lentivirus induced Oct4 and Sox2 (Supplemental Figure S2).

**Figure 2:**
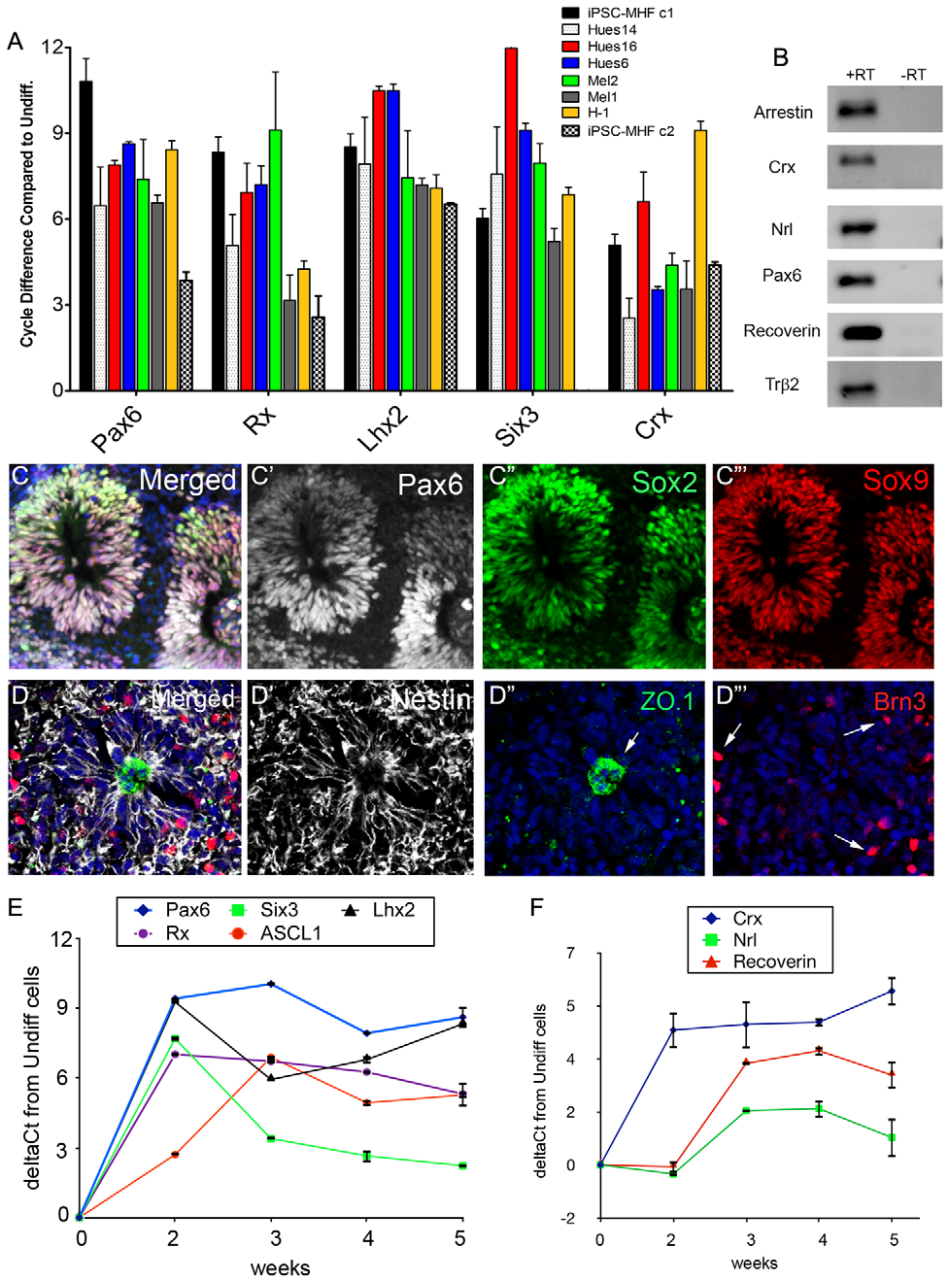
(A) RT-PCR comparison of expression of EFTFs and CRX in the various human ES and iPS cell lines expressed as a cycle change in PCR expression level compared to undifferentiated cells (n = 3–6) for the various lines, error bars represent SEM. (B) Gel showing expression of retinal markers by RT-PCR following retinal induction of iPS cells. (C) Co-expression of Pax6 (white, C, C′), Sox2 (green, C, C″), and Sox9 (red, C, C″′) by iPS cell derived retinal progenitors (merged in (C)) at the end of three weeks. The progenitors also expressed nestin (white, D,D') in the typical rosette pattern with ZO-1(green, D, D″) in the center and Brn3 expressing ganglion cells (red, D, D″′) at the periphery. (E, F) RT-PCR graph showing time course of expression of various retinal progenitor cell (E) and photoreceptor cell (F) genes over the course of 5 weeks. Error bars represent SEM.

Next we tested whether these cells could be directed to a retinal fate using a modification of our previously published protocol (see Methods). The undifferentiated iPS cells were plated on Matrigel-coated plates and treated with Dkk-1, IGF-1 and Noggin for three weeks. As described in our previous report [5], the efficiency of retinal determination was tested by analyzing the expression of several key eye-field transcription factors (EFTFs) using RT-PCR. All the EFTFs that we analyzed, Pax6, Six3, Lhx2 and Rx, showed a 6 to 10 cycle (80 to 1000 fold) increase over the undifferentiated cells at the end of two weeks of retinal induction (figure 2A). This is similar to that which we observed using the H1 human ES cell line (Lamba et al, 2006). In addition, there was on an average 5 cycle (34-fold) increase in expression of Crx at end of 2 weeks (figure 2A). We also analyzed the time course of the gene expression over 5 weeks of differentiation (Figure 2 E,F). We found that while the key EFTFs Pax6, Lhx2 and Rx showed a rapid and sustained up-regulation over the course of the experiment, the “late” progenitor marker, Ascl1 showed a much slower increase, with a peak at three weeks (Figure 2E). While these changes were similar to those we have previously reported from ES cells subjected to this protocol [5], the expression of Six3, another EFTF, was somewhat different in the iPSCs than what we have observed in the ES cells. Six3 shows an early peak, like the other EFTFs, but then declines over the next few weeks. We don't have an explanation for this difference, though it may be that ineffective silencing of the transgenes in the iPSCs (see above) may interfere with Six3 expression. We also analyzed the expression of the photoreceptor markers, Crx, recoverin and Nrl. All of these increased in the iPSCs as a function of time of treatment (Figure. 2F), albeit with somewhat different time courses. The pan-photoreceptor gene, Crx, showed early increases, while recoverin, a later photoreceptor marker of rods and cones, and Nrl, a rod photoreceptor transcription factor, increased after progressively longer periods of culture.

Since recent reports have shown substantial variability in the response of different ES and iPSC lines to directed differentiation protocols, we tested the effectiveness of this retinal determination protocol on a number of other human ES cell lines and one of the other iPS cell clone iPSC-MHF2 c2. These included three Harvard hES cell lines by Doug Melton's lab (Hues6, Hues14 and Hues16), and two Australian hES cell lines available through Millipore (Mel1 and Mel2). These cells were also cultured using the retinal determination protocol and analyzed for expression of the EFTFs at the end of two weeks. Overall, each of the lines responded to the induction protocol similarly to the H1 line; however, there were small variations in the expression levels of the EFTFs, as well as Crx, among the different cell lines. This was particularly evident in the case of the iPSC-MHF2 c2 line (Figure 2A), which showed much less effective induction of retinal genes than any of the other lines. Together with the results from the iPSCs, these data show the robustness of the protocol in creating retinal progenitors from various human ES as well as iPS cells.

**Figure 3:**
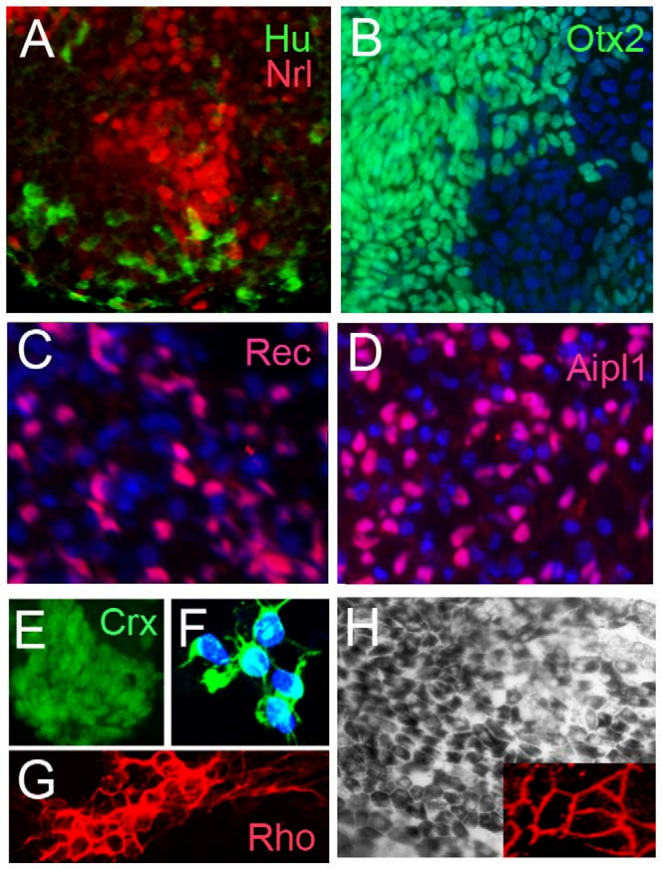
Cells in culture at two months after initiation of the protocol expressed HuC/D (green, A) marking ganglion and amacrine cells and Nrl (red, A) which labels rod photoreceptors. Photoreceptors were identified by the expression of Otx2 (green, B), Crx (E), recoverin (red, C) and AIPL1 (red, D). Cells also expressed differentiated markers like rhodopsin (red, G) and S-opsin (green, F). (H) Retinal pigmented epithelium was also generated from the iPS cells and these cells expressed ZO-1 (inset). Nuclei stained with DAPI in blue.

To confirm the RT-PCR analysis, we analyzed the retinal cells created from human iPS cells by immunocytochemistry for expression of various retinal markers. Retinal progenitors express Pax6, Sox2 and Sox9. We found that at the end of three weeks all three markers were co-expressed by the iPSCs that had been directed to a retinal fate (figure 2C-C″′). Overall by three weeks 70.22% (+/−5.24) of the cells expressed Pax6, 69.89% (+/−5.01) expressed Sox2 and 71.09% (+/−4.69) expressed Sox9. Most cells also expressed the neural progenitor marker nestin (figure 2D, D'). The culture plates also had patches of retinal pigmented epithelial cells, displaying pigmentation, characteristic hexagonal morphology and ZO-1 expression (figure 2D, D″, 3H). Other cells in the cultures expressed markers of inner retinal cell types: Hu C/D (figure 3A), which labels amacrine and ganglion cells and Brn3 which labels ganglion cells (figure 2D, D″′). We also analyzed the cells for markers for photoreceptor differentiation at the end of 2 months of induction. The cells in the retinal directed iPS cell cultures expressed the pan-photoreceptor markers Otx2 (9.6% (+/−1.13%)) (figure 3B) and Crx (11.8% (+/−2.9%)) (figure 3E). Cells also express the rod photoreceptor-specific transcription factor Nrl (figure 3A) and that 29.7% (+/−2.3%) of all photoreceptors were rod photoreceptors. Markers of more differentiated photoreceptors such as recoverin, AIPL-1, rhodopsin and S- opsin (figure 3C,D, G, F respectively) were expressed by less than 1% of cells at 2 months. These data were confirmed by PCR for expression of Crx, Nrl, arrestin, recoverin, Trβ2, rhodopsin and Pax6 (Figure 2B). Thus, human iPS cells can be induced into retinal fate and these cells express markers of retinal progenitors as well as differentiated cells such as ganglion cells, amacrine cells and photoreceptor cells.

**Figure 4:**
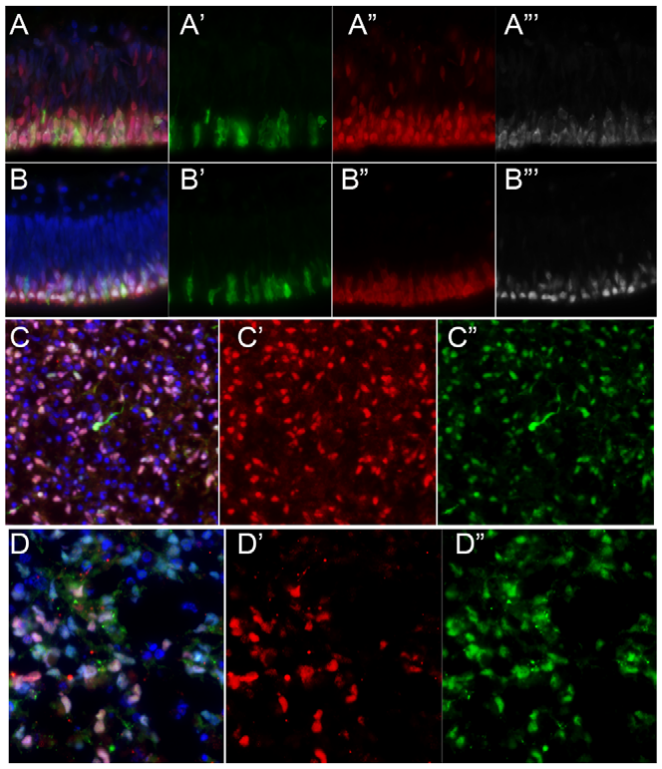
IRBP-GFP infection of human retinal explant resulted in GFP expression in photoreceptors as evidenced by co-expression of Otx2 (red, A, A″), AIPL1 (white, A, A″′), recoverin (red, B, B″) and Blimp1 (white, B, B″′). IRBP-GFP expression in iPS cell-derived retinal cells. GFP cells co-expressed AIPL1 (red, C, C'), Nrl (red, D, D'). Nuclei stained with DAPI in blue.

Ultimate cell-based therapy will require that the various types of retinal cells will need to be further purified from contaminating cell populations. One approach that has been used successfully in other regions of the nervous system is to use cell-type specific promoters coupled with fluorescent activated cell sorting (FACS). Inter-photoreceptor retinol binding protein (IRBP) is a photoreceptor specific gene expressed by both rod and cone photoreceptors early in their development prior to outer segment formation [14]. The human IRBP promoter has been shown to drive photoreceptor-specific expression in transgenic mice [15]. We constructed a lentivirus expressing GFP from the human IRBP promoter, and tested the specificity using human, mouse and chicken retinal explants. The IRBP-GFP lentivirus resulted in GFP expression only in the outer nuclear layer of all three species where the photoreceptors reside (Figure 4 and Supplementary Figure S1). Upon co-staining the human retinas for various photoreceptors markers including recoverin, Otx2, Blimp-1 and AIPL1, we found that GFP co-localized with photoreceptors in the retinas infected with IRBP-GFP (figure 4A–D). As a control for the specificity of infection, we used an EF1a lentivirus to infect other retinal explants; in these cases GFP was expressed in all of the various retinal cell types (Supplementary Figure S1). Thus, lentivirus with the human IRBP promoter driving GFP, specifically results in expression of GFP in photoreceptors of human, mouse and chicken retinas.

**Figure 5:**
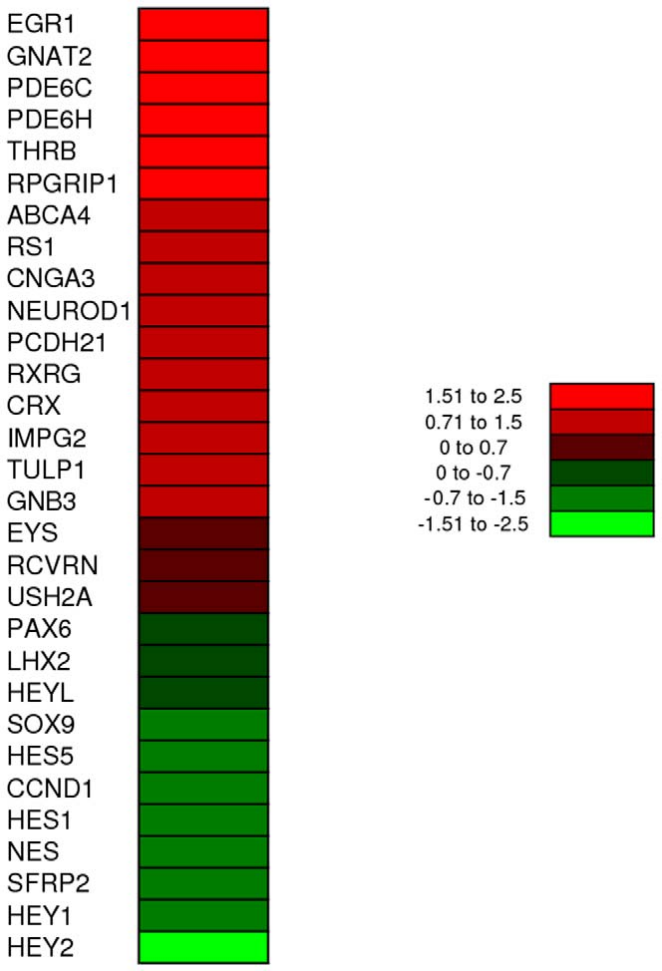
Microarray analysis of FACS sorted human fetal retinal IRBP-GFP cells and 96 day unsorted fetal retinal cells. The figure shows the heat map of the spot intensity comparison on the microarray of the various photoreceptor and retinal progenitor genes.

To test the ability to enrich photoreceptors, we infected 90 day fetal human retinal explants for 4 days in vitro and then dissociated the retinas. The dissociated cells were then subjected to fluorescent activated cell sorting (FACS). Following FACS, the cells were lysed in Trizol and RNA collected and used to run the Human Gene 1.0 ST array chip. We compared the results from the Affymetrix analysis of mRNA from FACS enriched photoreceptors to that from mRNA from a similarly staged (96 day) human fetal retina (GEO Accession # GSE18487). We found that many photoreceptor genes including PDE6C, GNAT2, PDE6H, THRB, RPGRIP1, CRX, ABCA4, RS1, TULP1, GNB3, EYS, IMPG2, PCDH21, USH2A, CNGA3, NEUROD1 and RCVRN were higher in the mRNA from the FACS purified photoreceptors, while progenitor genes like PAX6, LHX2, NESTIN, HES1, HES5, HEY1, HEY2 and SOX9 showed lower levels of expression when compared to mRNA from the 96 day fetal retina (figure 5). Thus, the microarray data confirmed that the IRBP-GFP resulted in photoreceptor specific GFP expression and that the cells can be enriched using FACS.

**Figure 6:**
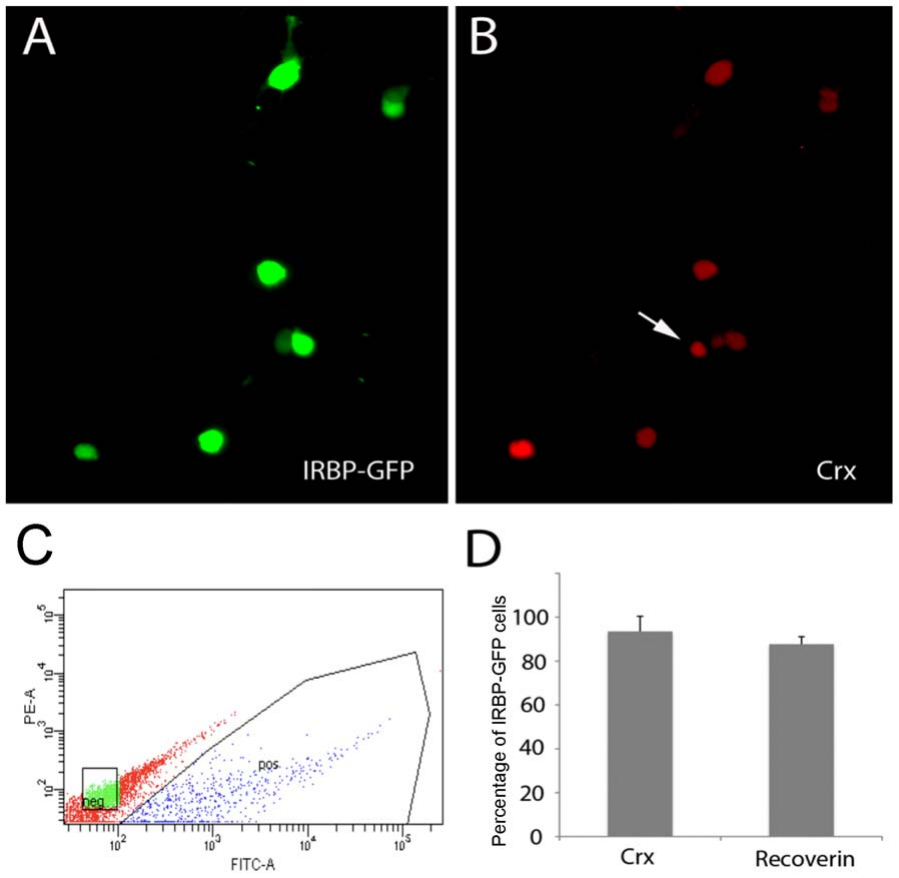
Fluorescent activated Cells sorting of human cells. Following FACS and fixation, upon immunostaining, GFP cells (A) expressed Crx (red, B). (C) Representative FACS plots for GFP cells in BD Aria II. (D) Plot showing percentage of sorted cells expressing Crx and recoverin.

Retinal cells derived from iPS cells or H-1 hES cells were then infected with the IRBP-GFP lentivirus 4 to 8 weeks after initiation of the retinal induction protocol. Based on our previous work using a similar backbone lentivirus, we expected 60–70% infection efficiency. We observed IRBP-GFP expressing cells in the culture plate from 4 days of infection and their numbers increased over the next few weeks in vitro. To confirm the identity of the IRBP-GFP expressing cells in the retinal cells derived from either hES cells or iPS cells, we fixed the cultures and processed them for immuno-fluorescent analysis with the photoreceptors markers AIPL1, rhodopsin, CRX and NRL (figure 4C-C′, D-D″). We found that nearly 100% of the GFP cells were labeled for CRX and the majority also expressed NRL, AIPL1 or rhodopsin. In order to purify the IRBP-GFP-expressing cells from the live iPS cell cultures, we subjected them to fluorescent-activated cell sorting (FACS) (figure 6C). On average, 10% of all cells in the cultures expressed IRBP-GFP. Following the FAC sorting, over 90% (92.18% +/−4.28) of the cells expressed the IRBP-GFP marker. Additionally, all the GFP+ cells stained for CRX and most for recoverin (figure 6A, B, D). Thus, retinal photoreceptors derived from iPS cells and H-1 hES cells can be enriched using a combination of IRBP-GFP lentivirus followed by FACS.

**Figure 7:**
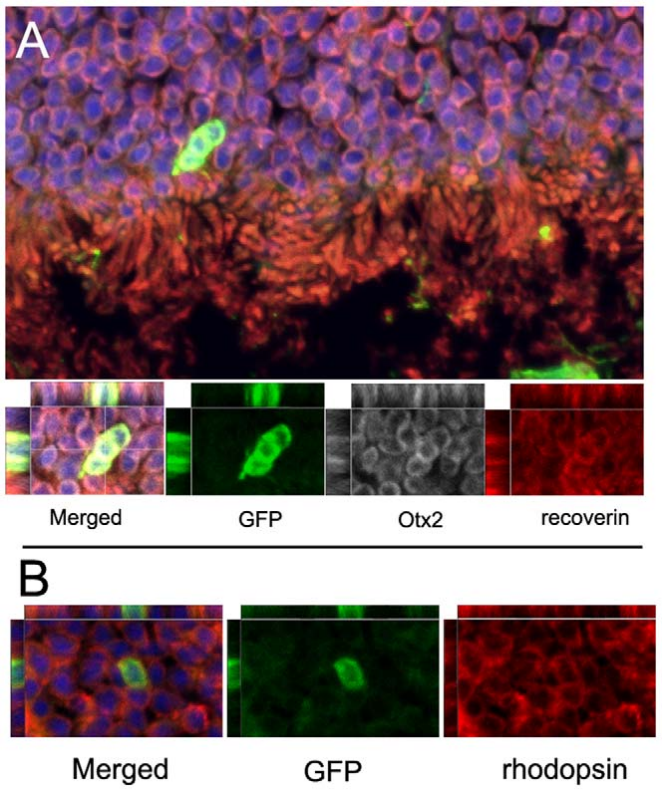
Transplantation of FACS IRBP-GFP human iPS cells inot wild-type mouse retina. (A) shows the photoreceptor layer stained for recoverin (red), Otx2 (white) and DAPI (blue). GFP expressing transplanted cells have migrated in and also express these markers. Also shown are the separate XY, XZ and YZ views of the three markers, GFP, Recoverin and Otx2 from confocal stack. (B) Shows a representative section of another example of an IRBP-GFP+ stained for GFP and rhodopsin (red) along with the XY, XZ and YZ views. Nuclei stained with DAPI in blue.

In our previous study, we found that retinal cells derived from human ES cells would migrate into the retina and differentiate into photoreceptors following transplantation to the sub-retinal space. To determine whether iPS cell-derived photoreceptors would incorporate into the retinas of mice following transplantation, we used a similar approach. iPS cells were directed to a retinal fate using our protocol and maintained in culture for an additional 4 weeks. The cells were then infected with the IRBP-GFP lentivirus and maintained in culture for an additional one to two weeks. Once a sufficient number of IRBP-GFP cells were present in the live cultures, we dissociated the cells and subjected them to FACS. The IRBP-GFP expressing population was then immediately transplanted into the sub-retinal space of adult wild-type mice. After a survival period of three weeks, the mice were euthanized and the eyes were removed, fixed and sectioned. The transplanted photoreceptors were identified using immuno-fluorescence for GFP and other photoreceptor markers. We found that iPS derived photoreceptors survived in the sub-retinal space over the three-week period, though at a much lower survival rate than the unsorted cells. We also found approximately 50 cells per eye that had migrated into the outer nuclear layer and were similar in appearance to hES derived photoreceptors following transplantation[2]. The iPSC-derived photoreceptors migrated well into the ONL and expressed the photoreceptor markers Otx2, recoverin and rhodopsin (figure 7A,B). These data further demonstrate the ability of human iPSCs to differentiate into photoreceptors, and further show that human iPS cell-derived photoreceptors can survive and integrate into the retina after FACS purification and transplantation.

## Discussion

In this report we have shown that human iPS cells can be used to generate retinal photoreceptors that can be purified by infecting with a lentivirus that drives GFP from the IRBP promoter and subsequent FACS. Our results show for the first time that human photoreceptors derived from either ES cells or iPS cells can be purified using a combination of photoreceptor-specific GFP vector and fluorescent activated cell sorting. Together with previous results that demonstrated the potential for ES cell derived photoreceptors to integrate following transplantation and restore light response to *CRX* −/− mice [2], the results presented in this report further support the possibility that stem cell approaches can lead to therapies for the treatment of retinal degenerations.

Three recent reports also show that other protocols used for retinal differentiation of ES cells can be used for iPS cells derived either from mouse or human to direct their differentiation to retinal cells [16], [17]. Hirami et al found that manipulations in Wnt and nodal pathways were able to induce retinal gene expression in 20% of their cells in 2 of the 3 human iPS cell lines they tested. They also showed that 14% of their colonies expressed Crx, though it is unclear how many cells in each colony expressed the marker. The same group also published another report using small molecules that affect the same molecular pathways and found a similar degree of retinal cell induction [18]. Meyer et al used a different approach of manually selecting floating spheres which had neural rosette morphology[17]. After manually selecting retinal spheres, ∼9% of the cells expressed Crx at 80 days, which is comparable to what we see from our protocol when we assay all cells in culture. In all cases, however, there is variability in the response of a particular iPSC line to the induction protocol. It will be interesting in the future to directly compare the same iPSCs with these different protocols to determine whether specific iPSC lines are not as responsive to differentiate as retinal cells, or alternatively, particular iPSC lines might be better suited to particular protocols of directed differentiation.

Human skin fibroblasts can be reprogrammed to a pluripotent state using several different methods [6], [8], [9], [19], [20]. Our results demonstrate that these reprogrammed fibroblasts can be directed towards a retinal progenitor pathway with efficiency similar to that of human ES cells. The presence of the pluripotency factors in vector proviruses does not appear to interfere with the differentiation of the cells to a retinal fate. In fact, we find that the protocol we developed for ES cells is as effective in directing the iPS cells to retinal cells as it is for many of the other ES lines we tested, even with incomplete silencing of the transgenes in the iPSCs. This is perhaps not surprising in light of recent evidence that iPSCs can differentiate into many different types of mature lineages [7], [12]. Nevertheless, more recent methods for deriving iPS cells do not rely on viral integration of the inducing genes and may therefore prove even more useful for the derivation of iPS cells for cell based therapies [21], [22], [23], [24].

Our findings may lead to the generation of human photoreceptors from individuals with inherited retinal degenerations, like Retinitis Pigmentosa (RP) and the development of additional models for these disorders. Although animal models have been generated for several forms of RP, there are many different mutations that cause this disease [25]. The ability to make photoreceptors from iPS cells should allow the generation of in-vitro models for these forms of RP. The iPSC-derived photoreceptors may therefore be useful for screening for compounds that will slow or prevent rod degeneration in these individuals. Although the photoreceptors generated from iPS cells or ES cells do not fully mature in vitro, the differentiation of ES cells can be facilitated through transplantation into the mouse retina, and a similar approach may be used for iPS cell derived photoreceptors. In addition to RP, iPS derived photoreceptors may be useful for developing therapies for patients with early onset retinal degenerations. Developmental disorders are particularly amenable to modeling with iPS cells. Recent studies have shown that motor neurons derived from iPS cells from patients with spinal muscular atrophy have been shown to have selective motor neuron death [26]. Photoreceptors derived from iPS cells from patients with Leber's congenital amaurosis or Usher's disease would be good candidates for a similar approach.

We have also found that the photoreceptors derived from ES cells and from iPS cells can be labeled in live cultures when infected with a lentivirus that drives GFP expression from the IRBP promoter sequences. The GFP expressing cells can be purified by FACS technology to contain nearly 90% photoreceptors, as assessed with subsequent labeling with rod and cone markers. The ability to purify the cells from undifferentiated contaminants is critical in developing a safe cell-based therapy for retinal degenerations. Earlier studies using less differentiated cell for transplantation have found evidence of teratoma formation; by comparison, we have never observed a teratoma following transplantation in over 100 mice to date with photoreceptors derived from ES cells using our directed differentiation protocol. A similar approach has been used to successfully reduce the risk of teratoma formation in transplantation of dopamine neurons derived from iPS cells [27]. Thus, using the FACS sorted photoreceptor cells will add an additional level of security against the risk of teratoma formation in cell based therapies using hES cells.

Photoreceptors derived from iPS cells can be transplanted and integrate into the retina. The iPSC-derived photoreceptors behaved very similarly to cells derived from human ES cells. After transplantation to the sub-retinal space, the cells begin to move into the retina of normal adult mice within a few weeks. Cells assume positions throughout the outer nuclear layer, and have protein expression pattern similar to mouse rod photoreceptors, labeling for Otx2, recoverin and rhodopsin. Our recent study has shown that photoreceptors derived from human ES cells were able to restore some light response to Crx −/− mice [2]. We found that FAC sorted cells did not survive as well as unsorted cells and so there were insufficient cells integrated into the retina for functional restoration. Nevertheless, the demonstration that iPS cells can develop into rods that survive and integrate after transplantation to the adult retina provides hope that autologous transplantation can be developed as a treatment for some forms of retinal degeneration. While gene therapy and medical therapies are being developed for the more common retinal degenerations, such as macular degeneration, there are millions of individuals with significant visual loss that would benefit from a cell-replacement therapy. Our results demonstrate the proof of principle that iPS cells derived from patients with retinal diseases may be useful in such therapies.

## Supporting Information

Figure S1 Lentivirus testing on mouse and chicken explant retinas. (A) shows GFP expression from the IRBP-GFP lentivirus in mouse retina in the photoreceptors as evidenced by co-staining with recoverin (red). (B) Control EF1a-GFP lentivirus resulted in GFP in all mouse retinal cells. (C), (D) show similar IRBP-GFP (C) in chicken photoreceptors as confirmed by photoreceptor marker visinin (red) while Ef1a-GFP (D) resulted in GFP in all chicken retinal cells.
(7.70 MB TIF)

Figure S2 PCR analysis to assess silencing of exogenous pluripotency genes. Above, gel image shows the expression of the 4 pluripotency genes used for the creation of the iPS cell lines at passage 12 of undifferentiated cell culture as well as 4 weeks following retinal induction. By the end of 4 weeks, Lin28 and Nanog were completely silenced, while Oct4 was reduced compared to undifferentiated cells while Sox2 was not silenced at all.
(0.38 MB TIF)
