# Supplementary figures and images for: Generation, Purification and Transplantation of Photoreceptors Derived from Human Induced Pluripotent Stem Cells
Source: PLoS One. 2010 Jan 20;5(1):e8763. doi: 10.1371/journal.pone.0008763 (PMC2808350; doi:10.1371/journal.pone.0008763)

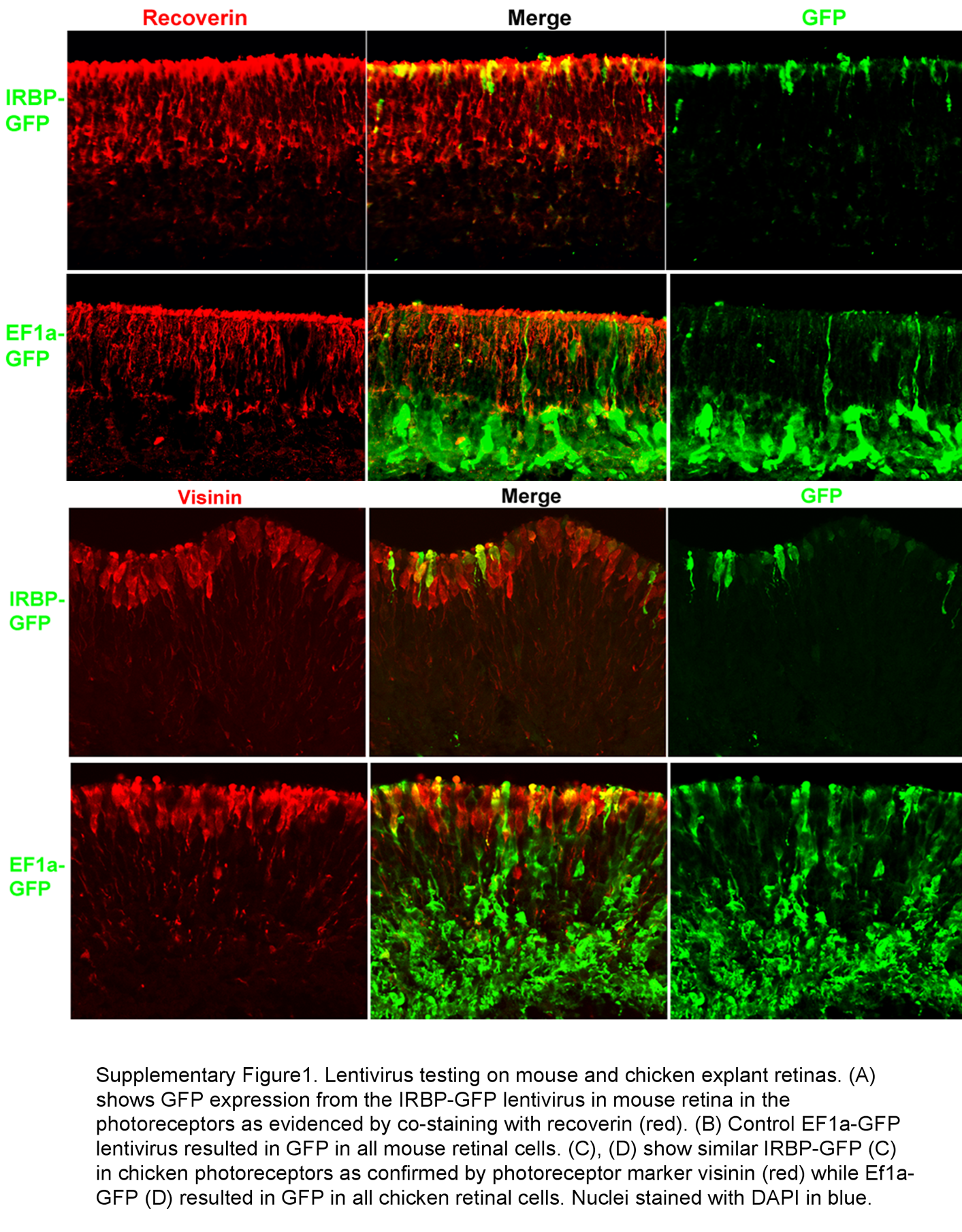

Supplement: Figure S1 — Lentivirus testing on mouse and chicken explant retinas. (A) shows GFP expression from the IRBP-GFP lentivirus in mouse retina in the photoreceptors as evidenced by co-staining with recoverin (red). (B) Control EF1a-GFP lentivirus resulted in GFP in all mouse retinal cells. (C), (D) show similar IRBP-GFP (C) in chicken photoreceptors as confirmed by photoreceptor marker visinin (red) while Ef1a-GFP (D) resulted in GFP in all chicken retinal cells. (7.70 MB TIF) [file pone.0008763.s001.tif]

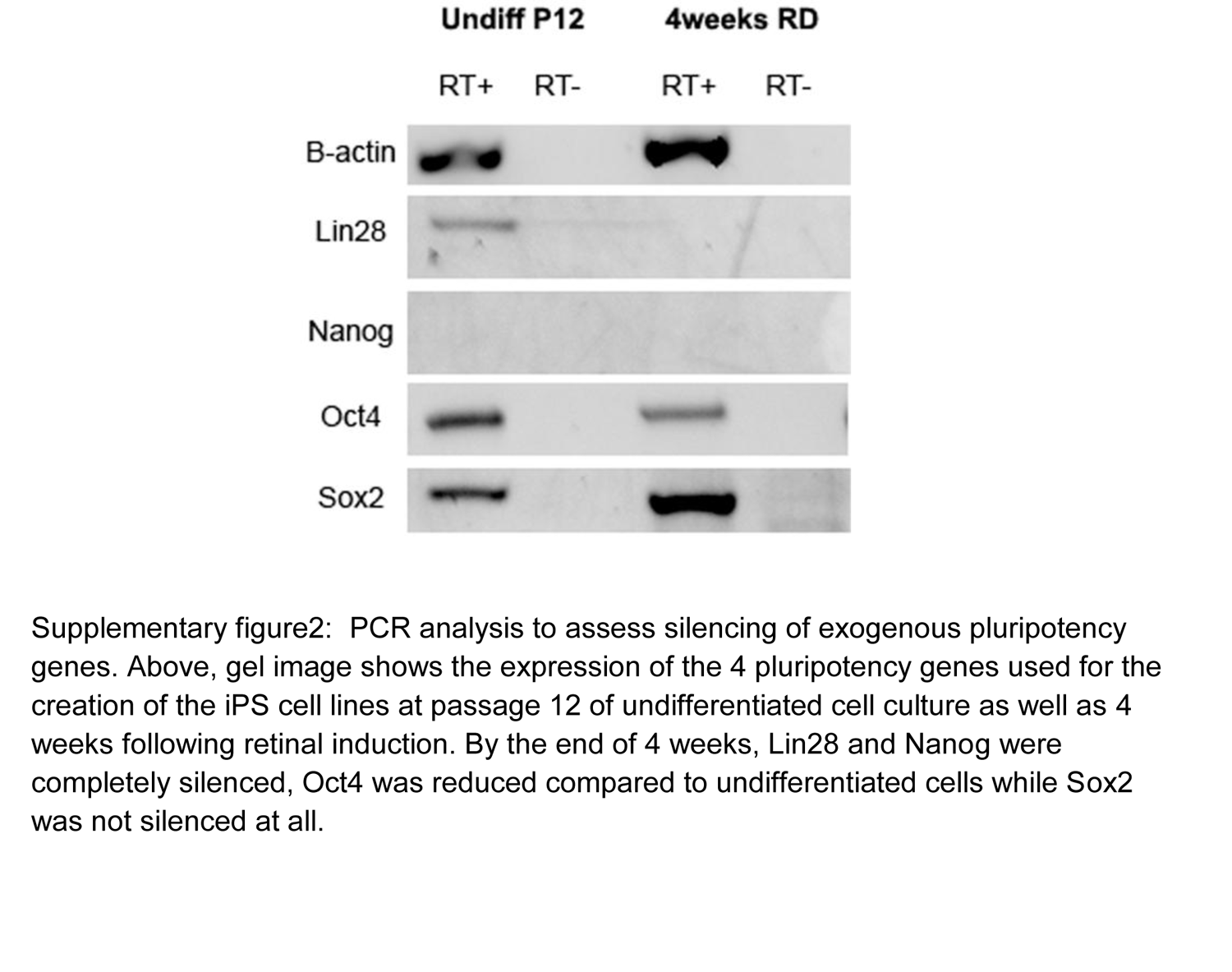

Supplement: Figure S2 — PCR analysis to assess silencing of exogenous pluripotency genes. Above, gel image shows the expression of the 4 pluripotency genes used for the creation of the iPS cell lines at passage 12 of undifferentiated cell culture as well as 4 weeks following retinal induction. By the end of 4 weeks, Lin28 and Nanog were completely silenced, while Oct4 was reduced compared to undifferentiated cells while Sox2 was not silenced at all. (0.38 MB TIF) [file pone.0008763.s002.tif]
